# Pretreatment brain volumes can affect the effectiveness of deep brain stimulation in Parkinson's disease patients

**DOI:** 10.1038/s41598-020-79138-9

**Published:** 2020-12-16

**Authors:** Younghee Yim, Sang Joon Kim, Seung Chai Jung, Ho Sung Kim, Choong Gon Choi, Jung Kyo Lee, Chong Sik Lee, Seung Hyun Lee, Woo Hyun Shim, E.-N. Cheong, Seong-Cheol Park

**Affiliations:** 1 Department of Radiology, College of Medicine, Chung-Ang University Hospital, Chung-Ang University, Seoul, Republic of Korea; 2 Department of Radiology, Asan Medical Center, University of Ulsan College of Medicine, 88 Olympic-ro 43-gil, Songpa-Gu, Seoul, 05505 Republic of Korea; 3 Department of Neurosurgery, Asan Medical Center, University of Ulsan College of Medicine, Seoul, Republic of Korea; 4 Department of Neurology, Asan Medical Center, University of Ulsan College of Medicine, Seoul, Republic of Korea; 5 Clinical Research Team, DEEPNOID Inc., Seoul, Republic of Korea; 6 Department of Neurosurgery, Hallym Hospital, Incheon, Republic of Korea

**Keywords:** Neurology, Neurological disorders, Parkinson's disease, Medical imaging, Brain imaging

## Abstract

We aimed to assess whether brain volumes may affect the results of deep brain stimulation (DBS) in patients with Parkinson’s disease (PD). Eighty-one consecutive patients with PD (male:female 40:41), treated with DBS between June 2012 and December 2017, were enrolled. Total and regional brain volumes were measured using automated brain volumetry (NeuroQuant). The Unified Parkinson Disease Rating Scale motor score quotient was used to assess changes in clinical outcome and compare the preoperative regional brain volume in patients categorized into the higher motor improvement and lower motor improvement groups based on changes in the postoperative scores. The study groups showed significant volume differences in multiple brain areas. In the higher motor improvement group, the anterior cingulate and right thalamus showed high volumes after false discovery rate (FDR) correction. In the lower motor improvement group, the left caudate, paracentral, right primary sensory and left primary motor cortex showed high volume, but no area showed high volumes after FDR correction. Our data suggest that the effectiveness of DBS in patients with PD may be affected by decreased brain volume in different areas, including the cingulate gyrus and thalamus. Preoperative volumetry could help predict outcomes in patients with PD undergoing DBS.

## Introduction

Deep brain stimulation (DBS) of the subthalamic nucleus (STN) has been proposed as a standard treatment for Parkinson’s disease (PD)^1^. However the cerebral networks targeted by DBS are poorly described and understood, and currently, there are no objective predictors of the post-treatment clinical response^2^. DBS of the STN in patients with PD improves not only motor symptoms, but also non-motor problems and levodopa-induced motor complications, which in turn improves in the overall quality of life of these patients^2^. Although the mechanism of action of DBS is unclear, it is hypothesized that complex modulation of the cortico-subcortical networks or the basal ganglia loops might be involved^3–5^. In a rodent model of PD, direct connections from the STN to the frontal cortex and M1 were necessary for the therapeutic effects of STN-DBS^6^. In a previous study, STN-DBS directly modified the firing probability of the corticofugal projection neurons in M1, leading to resolution of PD symptoms and improved motor control^7^. It is understood that STN-DBS may result in modulation of pathological oscillations in frontal brain networks through stimulation of the so-called hyperdirect pathway which is responsible for direct cortical projection to the STN^8^.

Previous studies have revealed associations between cortical thinning and aging^9^ and mild cognitive impairment in patients with PD^10^. However, the relationship between changes in brain volume and outcomes of DBS is unclear. Limited studies have been conducted to assess whether regional brain atrophy predicts the outcomes of DBS surgery, and inconsistent results have been reported. Muthuraman et al. have reported that the effects of STN-DBS in PD directly depend on frontal lobe grey matter integrity and suggested that cortical atrophy of this region may predict poor motor outcomes after STN-DBS in patients with PD^2^. Younce et al. have reported that thalamic and ventricular volumes predict motor response to DBS in PD^11^.

In this study we aimed to assess whether differences between regional brain volumes affects the outcomes of DBS in patients with PD. Therefore, we performed region-of-interest (ROI)-based volumetry using commercially available automated segmentation tool and validated the results using voxel-based volumetric analysis.

## Materials and methods

### Patients

**Figure 1:**
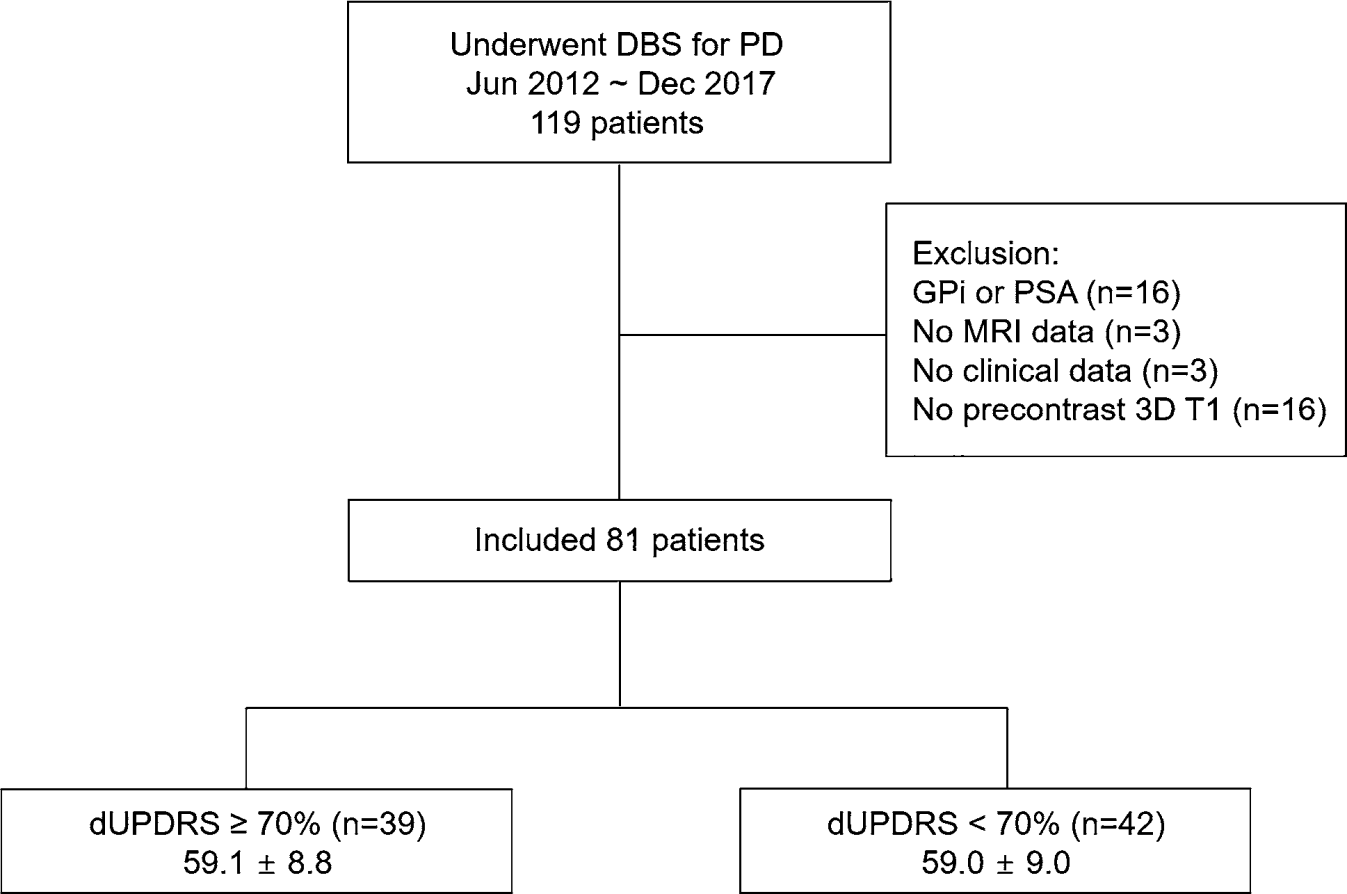
Patient flowchart.

The institutional review board (IRB) of Asan Medical Center approved this retrospective study, and the requirement for informed consent was waived. All procedures performed in the studies involving human participants were in accordance with the ethical standards of the institutional and/or national research committee and the 1964 Helsinki Declaration and its later amendments or comparable ethical standards. In total, 119 patients with clinically diagnosed PD who underwent DBS surgery for the management of movement disorders and high-resolution presurgical magnetic resonance imaging (MRI) between June 2012 and December 2017 were enrolled. Of the enrolled patients, 16 patients who underwent DBS surgery of the posterior subthalamic area or globus pallidus interna, 3 patients with no presurgical MRI data, 3 patients with no clinical follow-up data, and 16 patients with no pre-contrast three-dimensional T1 magnetization-prepared rapid gradient echo (MPRAGE) images were excluded from the study. The remaining 81 patients (male:female, 40:41; mean age, 59.0 [range, 35–74] years, who underwent DBS surgery of the STN were included (Fig. 1).

The diagnoses of PD according to established criteria were made by a neurologist specializing in movement disorders^12^. PD-related motor disability was assessed in both off- and on-medication states in all patients 1–2 months before DBS surgery, using Part III of the Unified Parkinson’s Disease Rating Scale (UPDRS)^13^. In all patients a postoperative UPDRS scores were also evaluated at 1-year follow-up. Postoperative UPDRS was measured for the following conditions: with both medication off, stimulation off and medication off, stimulation on.

Detailed information on the patient characteristics is provided in Table 1.

**Table 1:** Demographic data of the included patients.

	N = 81
Age (years)	59.0 ± 8.9
Male:female	40:41
Disease duration (year)	9.95 ± 4.6
**Preoperative UPDRS III scores**	
Medication OFF	50.1 ± 14.0
Medication ON	14.7 ± 9.8

Values reported as mean ± standard deviation.

### MRI acquisition

All MRI studies were performed on a 1.5-T scanner (Avanto, Siemens Healthcare, Germany) using an 8-channel head coil. High-resolution T1-weighted images (T1WI) of the brain was acquired using an MPRAGE sequence (repetition time, 7.7 ms; echo time, 3.6 ms; flip angle, 8°; 160 slices, slice thickness, 1 mm; matrix, 256 × 256 mm; isotropic resolution, 1 × 1 × 1 mm). The MRI DICOM files were uploaded to servers for processing.

### Surgical procedure

The surgical procedure consisted of a previously described MRI-guided method^14^. All the procedures were performed in accordance with relevant guidelines and regulations.

### Volume analysis

**Figure 2:**
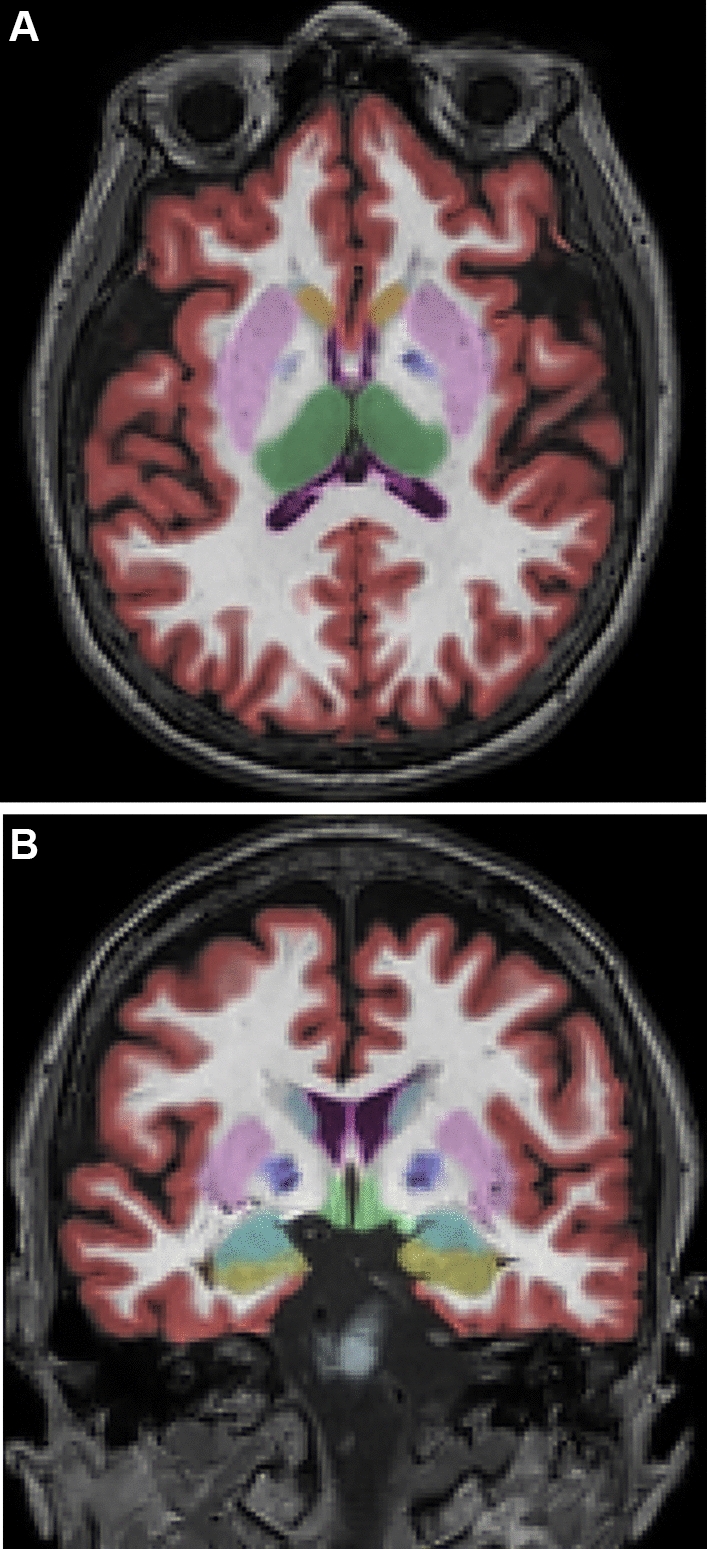
(**A**,**B**). Examples of automated color-coded segmentation results obtained with NeuroQuant. Light blue, caudate; pink, putamen; blue, pallidum; green, thalamus; cyan, amygdala; yellow, hippocampus; red, cortical grey matter.

Brain volume analysis was performed using a commercial software, NeuroQuant (CorTechs Labs, La Jolla, CA, USA, version 2.3, https://www.cortechslabs.com/products/neuroquant/), which received the US Food and Drug Administration 510 K clearance for clinical use to measure the volumes of brain structures on MRI. Automated MRI volumetry was performed using the standard NeuroQuant processing pipeline. The automated segmentation methods used by the software are based on widely used semiautomated methods^15,16^, and rely on probabilistic atlas-based methods to provide volumetric analysis of the segmented structures. Comprehensive details of the procedures are described elsewhere^17,18^. In brief, the protocol includes a quality check, adjustment for gradient non-linearity/B1 field inhomogeneity, skull stripping, and registration onto a probabilistic atlas using a discrete cosine transformation. An anatomic label is designated for each voxel based on the approximations from the probabilistic atlas. NeuroQuant uses a dynamic probabilistic neuroanatomical atlas, with age and gender specificity, based on the MR image intensity. The dynamic atlas is fitted to each subject by customizing the prior knowledge on each patient by generating a personalized atlas on the fly, based on the specific subject’s parameters, resulting in improved segmentation results over a broad continuous range of individuals. Details of the dynamic atlas are described elsewhere (https://www.cortechslabs.com/whitepapers/). The measurements comprise brain segmentation ROIs created by NeuroQuant (57 independent measurements and 9 combined or interpolated measurements). (https://www.cortechslabs.com/whitepapers/). Typical example of segmentation results obtained with NeuroQuant is provided (Fig. 2).

To validate the results obtained from automated volumetry, we also performed voxel-based morphometry (VBM) using SPM 12 (SPM; Wellcome Department of Cognitive Neurology; www.fil.ion.ucl.ac.uk). Images were preprocessed according to the guidelines, parameters, and templates recommended in the VBM tutorial (Ashburner, 2015). The T1-weighted MRI was segmented into grey matter (GM), white matter (WM), and CSF probability maps. These segmented GM and WM images were then spatially normalized to the customized template in the standardized anatomic space by using DARTEL (Wellcome Department of Imaging Neuroscience). To preserve the GM and WM volumes within each voxel, we modulated the images using the Jacobean determinants derived from the spatial normalization by DARTEL. Lastly, modulated images were smoothed using a 8-mm FWHM Gaussian kernel to increase the signal-to-noise ratio.

For the statistical analysis within SPM to confirm volume differences between groups, independent *t*-tests were used, including age, sex, and total brain volume as covariates; the total brain volume was calculated by summing the GM and WM volumes. Significance levels were set at *p* < 0.001 (uncorrected, k > 300). Montreal Neurological Institute (MNI) coordinates defining the anatomical regions of the clusters were also obtained.

### Clinical outcome analysis

The UPDRSIII motor score quotient was utilized to assess changes in clinical outcome as defined below:

$$dUPDRS\, = \,\frac{{\begin{array}{*{20}c} {{\text{Postoperative}} \,{\text{UPDRS}}\,{\text{medication}}\,{\text{OFF}},\, {\text{stimulation}}\, {\text{OFF}}} \\ { - {\text{Postoperative}}\, {\text{UPDRS}}\,{\text{medication}}\,{\text{OFF}},\,{\text{stimulation}}\,{\text{ON}}} \\ \end{array} }}{{{\text{Postoperative}}\,{\text{UPDRS}}\,{\text{medication}}\,{\text{OFF}},\,{\text{stimulation}}\,{\text{OFF}}}}\, \times \,100$$

The medication off state was defined as no medications for at least 12 h. The patients were categorized into the higher motor improvement (MI) group with dUPDRS ≥ 70 (n = 39) and the lower MI group with dUPDRS < 70 (n = 42) according to the dUPDRS score on the postoperative assessment.

### Evaluation of the electrode locations

The locations of the electrode were evaluated by the shortest distance between targeted coordinates and the measured coordinates of the active contact. The measured distance of the electrode was compared between the higher and lower MI group. A detailed description is included in the supplemental methods.

### Statistical analysis

Differences in the brain volume between the higher and lower MI groups were evaluated using univariate independent *t-*tests including age, sex, and intracranial volume as covariates. FDR correction was performed on ROIs that showed significant differences in the univariate analysis. Independent *t-*tests were used to compare demographical differences (i.e., age, percentage of males, disease duration) and changes in UPDRS score between groups. Logistic regression was used to identify the brain area showing the greatest influence on group differences. Pearson correlation analysis was performed between the brain areas and dUPDRS in all patients, with age, sex, and intracranial volume as covariates. A *p*-value of < 0.05 was considered to indicate a significant difference. All statistical analyses were performed using SPSS (IBM SPSS Statistics for Windows, version 21.0, IBM Corp.).

### Ethical approval and informed consent

All procedures performed in the studies involving human participants were in accordance with the ethical standards of the institutional and/or national research committee and with the 1964 Helsinki Declaration and its later amendments or comparable ethical standards. The requirement for informed consent was waived because of the retrospective nature of the study.

### Institutional approval

This study was pre-approved by the institutional review boards of Asan Medical Center (IRB No. 2016–0830).

## Results

### Motor outcomes after DBS

The demographic features of patients and the disease characteristics at baseline are summarized in Table 2. There were no statistically significant differences between the higher and lower MI groups in terms of age, sex, and disease duration. However, changes in UPDRS showed a statistically significant difference between the two groups (32.55 ± 25.83 in the lower MI group, 87.08 ± 9.51 in the higher MI group, *p* < 0.001). The dUPDRS in the higher MI group and in the lower MI group ranged from 70 to 100% and from − 50 to 66%, respectively. The shortest distance between the targeted coordinates and the measured coordinates of the active contact locations of the electrode tip was not significantly different between the two groups.

**Table 2:** Comparison of characteristics between the higher and lower MI groups.

	Higher MI group (n = 39)	Lower MI group (n = 42)	*p*
Age (years, mean ± standard deviation)	59.05 ± 8.83	58.95 ± 8.97	0.960
Percentage of male	59% (23)	40% (17)	0.098
Disease duration	9.85 ± 3.93	10.05 ± 5.16	0.845
Preoperative medication off	48.86 ± 12.99	51.33 ± 14.86	0.429
Preoperative medication on	13.08 ± 8.68	16.18 ± 10.69	0.157
Postoperative medication off stimulation on	4.2 ± 3.59	26.56 ± 16.93	0.000
Postoperative medication off stimulation off	30.53 ± 14.76	39.49 ± 19.42	0.023
Changes of UPDRS (dUPDRS)	87.08 ± 9.51	32.55 ± 25.83	0.000
Lead distance^a^, right (mm)	2.08 ± 1.11	2.17 ± 1.00	0.726
Lead distance^a^, left (mm)	2.06 ± 1.06	2.36 ± 1.16	0.229

Values reported as mean ± standard deviation.

MI,  motor improvement.

^a^The shortest distance between targeted coordinates and the measured coordinates of the active contact.

### Correlations between preoperative brain volumes and motor improvement

The higher and lower motor improvement groups showed volume differences in multiple brain areas. The anterior cingulate, right nucleus accumbens, left anterior middle frontal gyrus, and right thalamus showed significantly higher volumes in the higher MI group, while the left caudate, right primary sensory, paracentral, and left primary motor cortices showed a significantly lower volume. After FDR correction, the right nucleus accumbens and left anterior middle frontal gyrus did not show a significantly high volume; no area showed a significantly low volume.

The lower MI group tended to have smaller whole-brain volumes than the higher MI group (1186.392 ± 113.621 vs. 1260.495 ± 153.876), although the difference was not statistically significant (*p* = 0.471). Similar trends were also found at the individual lobar level, with the frontal, parietal, temporal, and occipital lobes showing greater brain volume in the higher MI group than in the lower MI group; however, the differences were also not significant. Similar findings were also noted at the gyrus level in the frontal lobe. Tables 3 and 4 show the results of the detailed volumetric comparisons between the two study groups.

**Table 3:** Comparison of regional brain volume between the higher and lower MI groups; areas with significant differences are listed.

	Higher MI group (n = 39)	Lower MI group (n = 42)	F	*p*	q
Anterior cingulate	7.304 ± 1.396	6.195 ± 0.873	12.245	0.001	0.008
R. nucleus accumbens	0.594 ± 0.111	0.518 ± 0.086	5.719	0.019	0.076
L. caudate	2.637 ± 0.59	2.687 ± 0.392	5.556	0.021	0.056
R. primary sensory	9.871 ± 2.311	9.887 ± 1.556	4.658	0.034	0.068
Paracentral	9.441 ± 1.971	9.666 ± 1.883	4.617	0.035	0.056
L. primary motor	10.565 ± 1.951	10.620 ± 1.533	4.200	0.044	0.058
L. anterior middle frontal	7.546 ± 1.557	6.634 ± 1.114	4.169	0.045	0.051
R. Thalamus	7.869 ± 1.001	7.259 ± 0.79	4.011	0.049	0.049

Values reported as mean ± SD.

MI,  motor improvement; R,  right; L,  left.

**Table 4:** Comparison of volume of the whole brain, each brain lobe, and frontal lobe between the higher and lower MI groups.

	Higher MI group (n = 39)	Lower MI group (n = 42)	F	*p*	q
**Whole brain**	1260.495 ± 153.876	1186.392 ± 113.621	0.524	0.471	0.753
Frontal lobe	172.263 ± 27.669	161.96 ± 19.427	0.051	0.821	0.938
Parietal lobe	111.883 ± 17.183	107.844 ± 12.321	0.235	0.629	0.838
Temporal lobe	129.061 ± 18.676	120.686 ± 12.085	1.746	0.190	0.760
Occipital lobe	52.214 ± 7.5	49.223 ± 7.576	0031	0.861	0.861
**Frontal lobe**	172.263 ± 27.669	161.96 ± 19.427	0.051	0.821	0.938
Superior frontal	47.216 ± 8.832	45.124 ± 6.747	0.620	0.434	0.868
Middle frontal	27.212 ± 4.857	24.816 ± 3.577	1.526	0.221	0.589
Inferior frontal	27.408 ± 4.431	25.279 ± 3.061	1.985	0.163	1.000

Values reported as mean ± standard deviation.

MI = motor improvement.

R = right, L = left.

In the logistic regression analysis of the areas showing significantly different brain volumes in 81 patients, the right thalamus showed the highest odds ratio, followed by the anterior cingulate gyrus, and left anterior middle frontal cortex (Table 5). In the correlation analysis, the volume of the anterior cingulate gyrus showed a significant correlation with dUPDRS scores (r = 0.246, *p* = 0.030) and the primary sensory cortex showed a significant negative correlation (r =  − 0.279, *p* = 0.013). In other areas, there was no significant correlation between dUPDRS and regional volume.

**Table 5:** Logistic regression results of significant difference areas of volume between the higher and lower MI groups.

Variable	B	S.E	Odds ratio	95% CI of odds ratio	
				Lower	Upper
R. Thalamus	1.527	0.626	4.603	1.349	15.707
Anterior cingulate	1.025	0.364	2.787	1.367	5.685
L. Anterior middle frontal	0.524	0.294	1.689	0.949	3.007
Paracentral	-0.264	0.226	0.768	0.493	1.195
R. Primary sensory	-0.391	0.261	0.676	0.405	1.129
L. Primary motor	-0.465	0.257	0.628	0.379	1.039
L. Caudate	-0.765	0.874	0.465	0.084	2.583

Odds ratio estimates for logistic regression model variables.

R = right, L = left.

**Figure 3:**
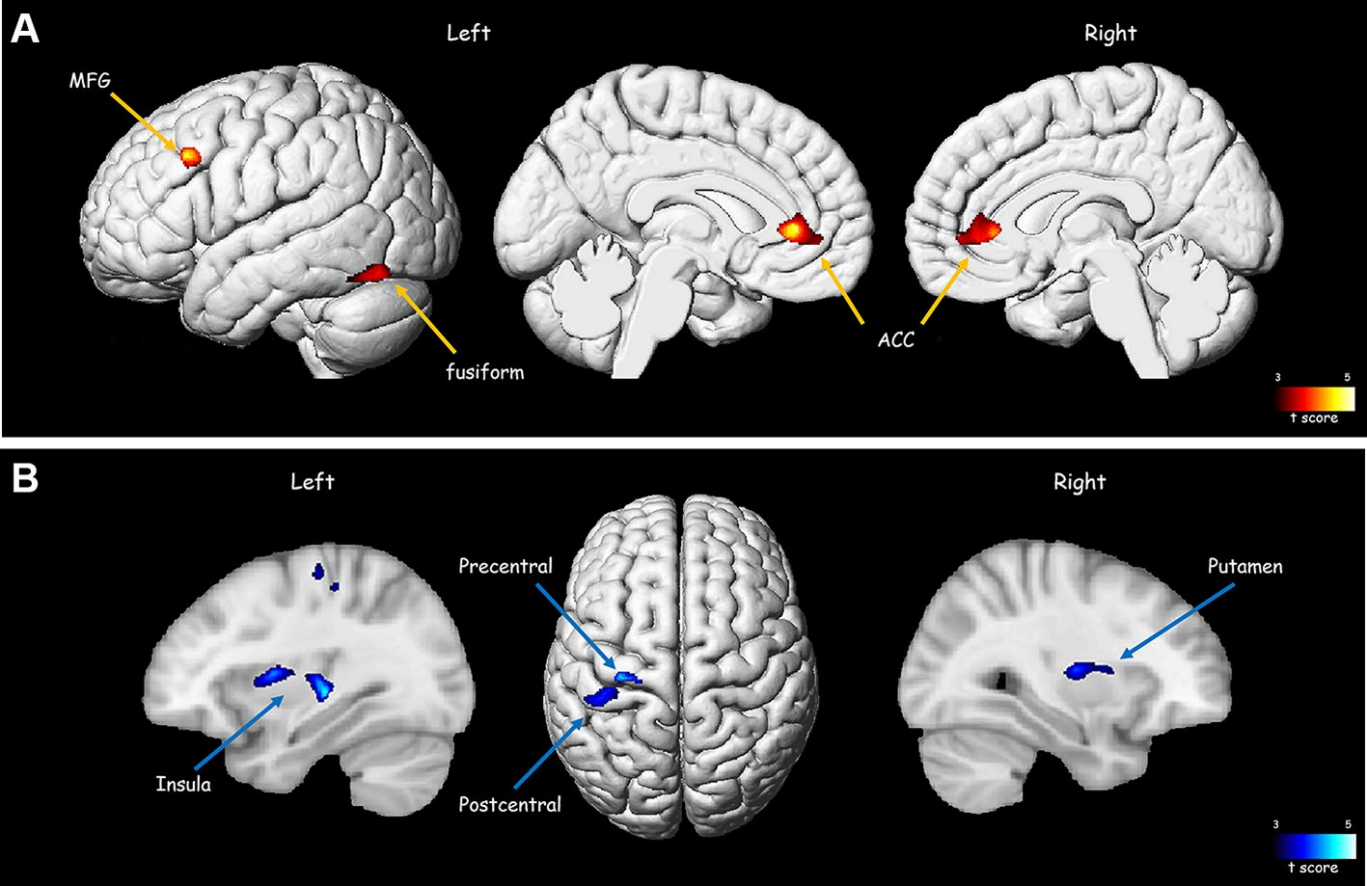
Illustrations of brain areas with significant grey matter volume differences between the higher and lower MI groups on VBM analysis. (**A**). Red areas represent higher volume in the higher MI group. (**B**). Blue areas represent higher volume in the lower MI group. (*p* < 0.001, k > 300). ACC = anterior cingulate cortex, MFG = middle frontal gyrus, VBM = voxel-based volumetry.

The VBM analysis showed significant volume differences between the higher and lower MI groups in the left middle frontal gyrus, right and left anterior cingulate gyrus, and the left fusiform gyrus (Fig. 3, Supplemental table). These results corresponded with the regions showing significant differences in the ROI-based analysis.

## Discussion

In this study, we used automated brain MRI volumetry (NeuroQuant) to investigate the correlation between preoperative brain volume and motor improvement after DBS for identifying the areas associated with potential motor improvement in patients with PD. Using anatomical three-dimensional T1WI, we found that the volumetry-derived volume measurements of several anatomical locations showed associations with the effects of DBS. When the higher MI group (change in UPDRS score; ≥ 70% improvement) were compared with the lower MI group (change in UPDRS score; < 70% improvement), there was a significant difference in the brain volume in the anterior cingulate, left anterior middle frontal gyrus, and right thalamus, with higher volumes in these areas in the higher MI group, but the left anterior middle frontal gyrus did not survive after FDR correction (q = 0.051). On the other hand, the right primary sensory, paracentral, left primary motor cortices and the left caudate showed lower volumes in the higher MI group than in the lower MI group, but they did not survive after FDR correction. Further, the total brain volume and brain volume of each cerebral lobe trended to be higher in the higher MI group than in the lower MI group; however, there was no statistically significant difference. We analyzed the frontal lobar volume in detail, as a previous study revealed that cortical atrophy of the paracentral and superior frontal areas may be associated with inferior clinical outcomes of DBS compared with intact cortical morphology^2^. Our study also revealed high volume areas in the frontal lobe in the higher MI group; however, the locations differed; the left anterior middle frontal gyrus showed high volumes in our higher MI group with borderline significance after FDR correction.

Previous studies have reported grey matter losses in sub-cortical structures such as the thalamus, caudate, and putamen in patients with PD and that cortical integrity remained preserved until later disease stages^2^. In another study using VBM, global grey matter loss, amygdala atrophy, and cortical thinning in the fronto-temporal regions were associated with the PD-degenerative process in patients with early PD^19^. Although some studies suggest that cortical thinning is only detected in patients with PD and neurocognitive or executive impairment^10,20^, Muthuraman et al.^2^ have recently reported that the neurodegenerative process reflected by cortical thinning is a pathophysiologic-relevant factor observed in patients with PD. According to Muthuraman et al., the integrity of the frontal cortical region, which is the main part of the motor network, determines the outcome of DBS in parkinsonian patients, possibly driven by cross-talk through the hyperdirect pathway from the stimulation site in the STN. Thus, they concluded that cortical atrophy of the frontal cortical region might represent a distinct predictor of a poor motor outcome after STN-DBS in patients with PD.

A recent study investigating the correlation between brain metabolism and movement improvement in patients with PD showed that increased M1 metabolism and/or decreased parieto-occipital metabolism were associated with less favorable motor outcomes after DBS^21^. They suggested that a PD-related motor pattern is associated with the capacity for motor improvement after DBS. Other studies have revealed increased glucose consumption in the putamen, STN, globus pallidus interna, thalamus, M1, brainstem, and cerebellum in patients with PD; these areas are known to be involved in motor execution. Further, in these studies significant metabolic reduction was noted in the parieto-occipital and temporal and frontal associative areas^22–26^. Some fMRI studies have also reported similar results to the FDG-PET studies. In our study, the primary sensory and motor and paracentral cortices showed lower volumes in the higher MI group; however they did not survive after FDR correction. This may be attributable to the relatively small number of included patients.

Younce et al. reported that thalamic volumes predict motor response to DBS in PD^11^. Our study also revealed high thalamic volume in the higher MI group. As the ventroanterior and ventrolateral nuclei of the thalamus are major downstream outputs of the STN and internal globus pallidus, structural changes in the thalamus may also affect the functional pathways by which DBS exerts its therapeutic effects^27^.

In our study, the cingulate gyrus showed a significantly high volume in the higher MI group than in the lower MI group, a result that was also found in the VBM analysis (Fig. 3). Logistic regression showed high odds ratio in the cingulate gyrus, and in the correlation analysis, there was a significant correlation between the volume of the cingulate gyrus and the dUPDRS. Loss of integrity and atrophy of the cingulate gyrus have been reported in PD, and are known to be related to cognitive symptoms^28^. In a recent study using diffusion tensor imaging, the preoperative connectivity of a network encompassing the frontal cortex, prefrontal cortex, and cingulate gyrus was directly linked to postoperative clinical outcomes^29^. We speculate that the condition of the cingulate gyrus is also related to the integrity of the connection between the STN and frontal lobe.

Our results are partially in line with a previous studies reporting associations between preoperative brain changes in PD and the outcomes of DBS surgery^2,11,21^. In previous reports, the results of preoperative evaluations of the brain by MRI or FDG-PET largely corresponded with each other, although different details were revealed. An MRI volumetry study showed frontal lobe atrophy change and atrophy of the thalamus and ventricles, while FDG-PET showed decreased metabolism in associative areas and in the parieto-occipital lobe in the group of worst responders. These differences may be caused by variation in the implemented tools, evaluation criteria, or patient groupings. A further study with a larger number of patients is needed for more reliable results.

In this study, we used ROI-based volumetry to evaluate volume differences between higher and lower MI groups after DBS surgery for PD. We used automated segmentation tools for ROI-based volumetry to obtain clinically feasible results and facilitate results for each individual patient. We also performed VBM analysis in the same groups to validate the results obtained from the ROI-based volumetry, and found that the two sets of results corresponded well.

The present study has several limitations. First, as this study was retrospective design and evaluated patients from a single center, a selection bias may exist. Second, grouping patients based on the criteria of 70% motor improvement in dUPDRS was arbitrary. We tried several other grouping methods to simulate the result, including the criteria of 30%, 50%, and 60% improvement in dUPDRS; however, these methods resulted in inconsistent and subtle differences between the higher and lower MI groups. Therefore, we chose the criteria of 70% improvement to maximize potential differences. This may have caused some bias in the results; however, the correlation between the volume of the cingulate gyrus and the dUPDRS results in the entire patient population supports our result. Third, in VBM analysis, there was no significant areas of volume difference between the higher and lower MI groups when performing FDR correction; therefore, there is a possibility of a false-positive error in our results. However, a level of uncorrected *p* > 0.001 was allowed in prior brain imaging studies. The reason for the lack of observed significant region after FDR correction may be attributable to the relatively small number of included patients, which may have hampered the sensitivity of the statistical analysis. Fourth, other compounding factors that may have influenced the results, such as disease duration or medication were disregarded. Future prospective large-scale studies are required to verify the correlations between regional cerebral atrophy and DBS efficacy in patients with PD, and the consistency of the results in patients treated with DBS.

## Conclusion

In conclusion, our data suggest that the effectiveness of DBS in patients with PD may be affected by decreased brain volume in different areas, including the cingulate gyrus and thalamus. We expect that evaluation of patients with PD with preoperative volumetry may help predict the outcomes of DBS.

## Supplementary Information

Supplementary information.

## References

[1] Volkmann J (2013). Selecting deep brain stimulation or infusion therapies in advanced Parkinson’s disease: an evidence-based review. J. Neurol..

[2] Muthuraman M (2017). Effects of DBS in parkinsonian patients depend on the structural integrity of frontal cortex. Sci. Rep..

[3] Lozano AM, Dostrovsky J, Chen R, Ashby P (2002). Deep brain stimulation for Parkinson's disease: disrupting the disruption. Lancet Neurol..

[4] Montgomery EB, Gale JT (2008). Mechanisms of action of deep brain stimulation (DBS). Neurosci. Biobehav. Rev..

[5] McIntyre CC, Hahn PJ (2010). Network perspectives on the mechanisms of deep brain stimulation. Neurobiol. Dis..

[6] Gradinaru V, Mogri M, Thompson KR, Henderson JM, Deisseroth K (2009). Optical deconstruction of parkinsonian neural circuitry. Science.

[7] Li Q (2012). Therapeutic deep brain stimulation in Parkinsonian rats directly influences motor cortex. Neuron.

[8] Brunenberg EJ (2012). Structural and resting state functional connectivity of the subthalamic nucleus: identification of motor STN parts and the hyperdirect pathway. PLoS ONE.

[9] Salat DH (2004). Thinning of the cerebral cortex in aging. Cereb. Cortex..

[10] Hanganu A (2014). Mild cognitive impairment is linked with faster rate of cortical thinning in patients with Parkinson’s disease longitudinally. Brain.

[11] Younce JR, Campbell MC, Perlmutter JS, Norris SA (2019). Thalamic and ventricular volumes predict motor response to deep brain stimulation for Parkinson's disease. Parkin. Relat. Disord..

[12] Hughes AJ, Daniel SE, Kilford L, Lees AJ, Neurosurgery & Psychiatry (1992). Accuracy of clinical diagnosis of idiopathic Parkinson's disease: a clinico-pathological study of 100 cases. J. Neurol. Neurosurg. Psychiatry..

[13] Fahn, S., Elton, R. & Committee. *Recent developments in Parkinson’s disease.* Vol. 2.0 53-163, 293–304 (MacMillan Healthcare Information, 1987).

[14] Park S-C, Lee CS, Kim SM, Choi EJ, Lee JK (2017). Comparison of the stereotactic accuracies of function-guided deep brain stimulation, calculated using multitrack target locations geometrically inferred from three-dimensional trajectory rotations, and of magnetic resonance imaging-guided deep brain stimulation and outcomes. World Neurosurg..

[15] Dale AM (2000). Dynamic statistical parametric mapping: combining fMRI and MEG for high-resolution imaging of cortical activity. Neuron.

[16] Fischl B (2002). Whole brain segmentation: automated labeling of neuroanatomical structures in the human brain. Neuron.

[17] Brewer JB, Magda S, Airriess C, Smith ME (2009). Fully-automated quantification of regional brain volumes for improved detection of focal atrophy in Alzheimer disease. AJNR Am. J. Neuroradiol..

[18] England HB, Gillis MM, Hampstead BM (2014). RBANS memory indices are related to medial temporal lobe volumetrics in healthy older adults and those with mild cognitive impairment. Arch. Clin. Neuropsychol..

[19] Ibarretxe-Bilbao N (2012). Progression of cortical thinning in early Parkinson's disease. Mov. Disord..

[20] Jubault T (2011). Patterns of cortical thickness and surface area in early Parkinson's disease. Neuroimage.

[21] Lee EJ (2019). Parkinson disease-related pattern of glucose metabolism associated with the potential for motor improvement after deep brain stimulation. Neurosurgery.

[22] Nagano-Saito A (2004). Cognitive-and motor-related regions in Parkinson's disease: FDOPA and FDG PET studies. Neuroimage.

[23] Huang C (2013). Neuroimaging markers of motor and nonmotor features of Parkinson's disease: an [18F] fluorodeoxyglucose positron emission computed tomography study. Dement. Geriatr. Cogn. Disord..

[24] Arahata Y (1999). Parieto-occipital glucose hypometabolism in Parkinson’s disease with autonomic failure. J. Neurol. Sci..

[25] Huang C (2007). Changes in network activity with the progression of Parkinson's disease. Brain.

[26] Ma Y, Tang C, Spetsieris PG, Dhawan V, Eidelberg D (2007). Abnormal metabolic network activity in Parkinson's disease: test—retest reproducibility. J. Cereb. Blood Flow Metab..

[27] Benazzouz A (2000). Effect of high-frequency stimulation of the subthalamic nucleus on the neuronal activities of the substantia nigra pars reticulata and ventrolateral nucleus of the thalamus in the rat. Neuroscience.

[28] de Schipper LJ, van der Grond J, Marinus J, Henselmans JM, van Hilten JJJNC (2017). Loss of integrity and atrophy in cingulate structural covariance networks in Parkinson's disease. Neuroimage Clin..

[29] Koirala N (2018). Frontal lobe connectivity and network community characteristics are associated with the outcome of subthalamic nucleus deep brain stimulation in patients with Parkinson’s disease. Brain Topogr..

