# Supplementary material for: Pretreatment brain volumes can affect the effectiveness of deep brain stimulation in Parkinson's disease patients
Source: Sci Rep. 2020 Dec 16;10:22065. doi: 10.1038/s41598-020-79138-9 (PMC7744532; doi:10.1038/s41598-020-79138-9)
Supplement: Supplementary file 1 — Supplementary information. [file 41598_2020_79138_MOESM1_ESM.pdf]

Supplemental Methods

Evaluation of the electrode locations

To evaluate the anatomical localization of the active contact and tips of electrodes, postoperative CT with 1-mm slice thickness was obtained in all patients. The coordinates of electrodes were measured using Framelink software (Medtronic Inc., Minneapolis, Minnesota, USA).

Lead locations were measured as a continuous variable in three dimensions with respect to the midpoint of the intercommissural line. Stereotactic accuracy was calculated by the shortest distance between the targeted coordinates and the measured coordinates of the active contact.

Supplemental table. Brain areas with significant volume differences on VBM analysis using SPM ($p < 0.001$, $k > 300$)

Brain Areas	No. of voxels	MNI coordinates (mm)			t-value
		x	y	z	
**Higher MI group > Lower MI group**					
Left Middle frontal gyrus	809	-34	19	36	4.36
Left Anterior cingulate gyrus	1590	0	35	4	4.21
Right Anterior cingulate gyrus		2	46	-1	3.43
Left Fusiform gyrus	2066	-42	-67	-17	4.20
		-38	-47	-16	4.08
**Higher MI group < Lower MI group**					
Left insula	2971	-32	1	9	4.09
		-43	2	0	3.23
Left Postcentral gyrus	637	-44	-33	53	3.89
Right Putamen	1262	31	-10	10	3.87
		33	4	8	3.60
Left Precentral gyrus	341	-30	-21	59	3.53
		-21	-23	66	3.25

MI = motor improvement, VBM = voxel-based volumetry
